# Patient access to chronic medications during the Covid-19 pandemic: Evidence from a comprehensive dataset of US insurance claims

**DOI:** 10.1371/journal.pone.0249453

**Published:** 2021-04-01

**Authors:** Jeffrey Clement, Maura Jacobi, Brad N. Greenwood

**Affiliations:** 1 Information and Decision Sciences, University of Minnesota, Carlson School of Management, Minneapolis, Minnesota, United States of America; 2 United Family Medicine Residency, Allina Health, St. Paul, Minnesota, United States of America; 3 Information Systems & Operations Management, George Mason University, School of Business, Arlington, Virginia, United States of America; University of Manitoba, CANADA

## Abstract

Patient access and adherence to chronic medications is critical. In this work, we evaluate whether disruptions related to Covid-19 have affected new and existing patients’ access to pharmacological therapies without interruption. We do so by performing a retrospective analysis on a dataset of 9.4 billion US prescription drug claims from 252 million patients from May, 2019 through August, 2020 (about 93% of prescriptions dispensed within those months). Using fixed effect (conditional likelihood) linear models, we evaluate continuity of care, how many days of supply patients received, and the likelihood of discontinuing therapy for drugs from classes with significant population health impacts. Findings indicate that more prescriptions were filled in March 2020 than in any prior month, followed by a significant drop in monthly dispensing. Compared to the pre-Covid era, a patient’s likelihood of discontinuing some medications increased after the spread of Covid: norgestrel-ethinyl estradiol (hormonal contraceptive) discontinuation increased 0.62% (95% CI: 0.59% to 0.65%, p<0.001); dexmethylphenidate HCL (ADHD stimulant treatment) discontinuation increased 2.84% (95% CI: 2.79% to 2.89%, p<0.001); escitalopram oxalate (SSRI antidepressant) discontinuation increased 0.57% (95% CI: 0.561% to 0.578%, p<0.001); and haloperidol (antipsychotic) discontinuation increased 1.49% (95% CI: 1.41% to 1.57%, p<0.001). In contrast, the likelihood of discontinuing tacrolimus (immunosuppressant) decreased 0.15% (95% CI: 0.12% to 0.19%, p<0.001). The likelihood of discontinuing buprenorphine/naloxone (opioid addiction therapy) decreased 0.59% (95% CI: 0.55% to 0.62% decrease, p<0.001). We also observe a notable decline in new patients accessing these latter two therapies. Most US patients were able to access chronic medications during the early months of Covid-19, but still were more likely to discontinue their therapies than in previous months. Further, fewer than normal new patients started taking medications that may be vital to their care. Providers would do well to inquire about adherence and provide prompt, nonjudgmental, re-initiation of medications. From a policy perspective, opioid management programs seem to demonstrate a robust ability to manage existing patients in spite of disruption.

## Introduction

The SARS-CoV-2 coronavirus pandemic has disrupted supply chains globally and impacted millions of people who rely on uninterrupted access to medications for chronic conditions. In this study, we evaluate whether patients in the United States were able to maintain access to selected therapies during the Covid-19 pandemic.

A culmination of stay-at-home orders, social distancing requirements, financial pressures, school closures, and increased personal stress raise the possibility of refilling medication becoming a lower priority, or an impossibility, due to these unprecedented roadblocks. With more than 7.3 million workers losing their health insurance, and medical facilities limiting hours for routine visits or closing altogether, there has been a 40% decline in outpatient visits since March 2020 [1, 2]. Supply chain issues and manufacturer backorders further complicates matters. Although pharmacies discouraged patients from stockpiling medication to avoid waste and shortages, the American Society of Health-System Pharmacists estimates there was an 8% increase in the number of active drug shortages during the first quarter of 2020 [3].

While these figures are troubling, what remains unclear is which patients and which therapies, if any, the Covid-19 pandemic has substantively interrupted. Patients may have, for example, switched to a mail order pharmacy or received longer than 90-day refills. For patients unable to physically visit their doctor, telehealth visits have emerged as a viable alternative to support patients who may need a prior authorization, a refill or a new prescription. While many insurance providers and pharmaceutical benefit managers (PBMs) place limits on how early patients may refill their medications (e.g., 2–7 days before the expected runout date), many payers relaxed these restrictions in response to Covid-19, and the US Drug Enforcement Administration provided updated guidance on early refills [4]. Our objective is therefore to determine the extent and degree to which filling and denial patterns changed across cornerstone drugs unimpacted by shortage during Covid-19 in six therapeutic classes with significant population health impacts (viz. addiction, immunosuppression, hormonal contraception, stimulants for ADHD, SSRIs for mood disorders, and antipsychotics).

## Materials and methods

### Dataset

Data are drawn from Symphony Health (SH), a health sciences data firm. SH aggregates from a variety of sources, particularly insurance claims clearinghouses and pharmacies, and estimates they capture 93% of the prescriptions dispensed in the US [5]. Data covers all payer types, including cash/uninsured patients, and is matched at the patient level. This allows us to track patients longitudinally even if they switch insurers or pharmacies. This dataset is regularly used in medical, regulatory, and policy research, as well as by commercial firms like pharmaceutical and insurance companies [e.g., 6–9]. The extract from this dataset that we received contains de-identified data on 9.4 billion prescription claims from more than 252 million patients between May, 2019 and August, 2020.

### Drug selection

The reported drugs are intended to represent a selection of important chronic medications and the identified usage/refilling patterns. In each category, we selected a major drug with an available generic *a priori*. Drugs were selected based on the authors’ familiarity and review of literature in each domain, but it should be noted that patient needs and care settings vary considerably. As a result, the observed trends for one drug are not determinative of all other drugs in the same category (discussed further below). These drugs include buprenorphine/naloxone (oral film and tablets for addiction), tacrolimus (immunosuppressant), norgestrel-ethinyl estradiol (hormonal contraceptive), dexmethylphenidate HCL (a stimulant for ADHD), escitalopram oxalate (an SSRI for depression), and haloperidol (an antipsychotic). These drugs did not experience novel shortages as a result of Covid-19; see S1 Table for details. S2 Table contains summary statistics of the dataset by drug. We only include claims for the buprenorphine/naloxone combination medication, originally released under the trade name Suboxone, rather than either drug sold independently. We consider all branded and generic formulations, including both film and tablet formulations. We do not consider single-molecule buprenorphine formulations. In the case of tacrolimus, we include only oral tacrolimus for immunosuppression, screening out the topical formulations often used for eczema.

### Model free evidence

**Fig 1:**
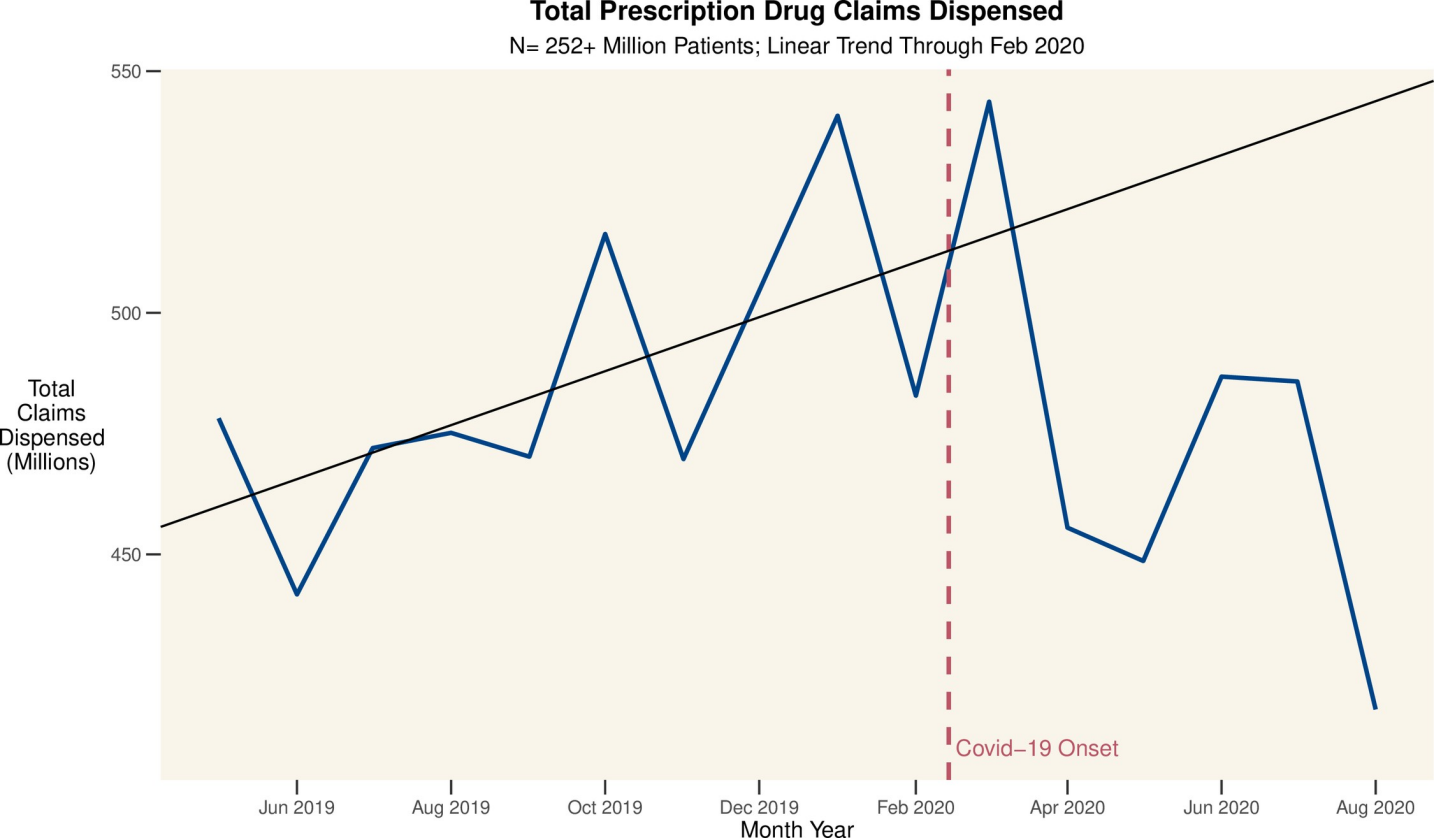
US prescription drug claims dispensed by month. The figure shows the total number of approved pharmaceutical claims in the database; the red line indicates the ramp up of Covid-19 cases and response in the US. The trendline is based on data from May 2019 through February 2020 and represents a linear forecast for the period beyond February 2020. There were 12.05% fewer claims in August 2020 than in August 2019, likely due a combination of factors: stock up in March 2020, reduced demand (due to delayed elective procedures), and access challenges.

**Fig 2:**
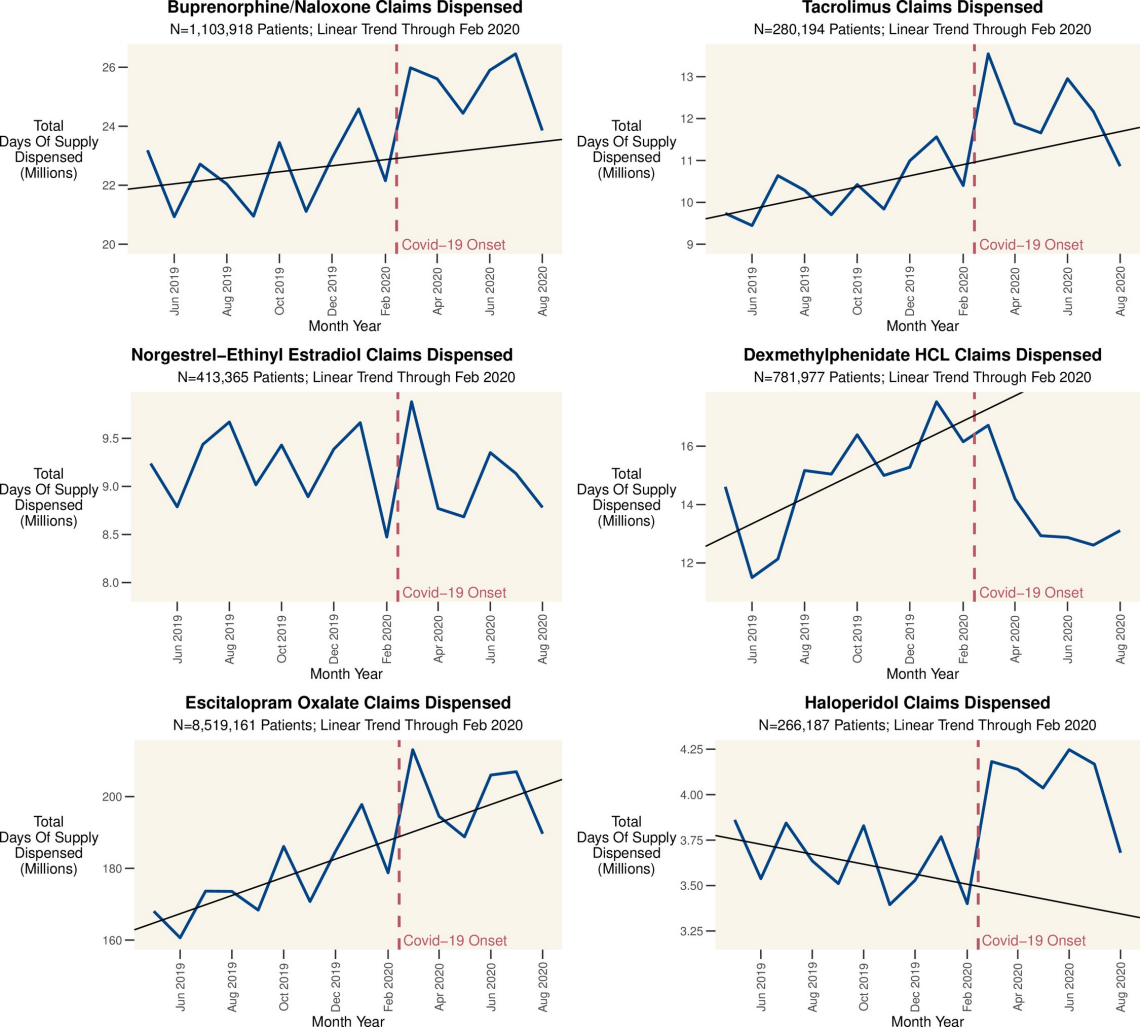
Total days of supply (DOS) dispensed by month across key drugs. Total days of supply (DOS) dispensed by month across key drugs. March 2020 represents the peak demand for many drugs, though there are exceptions such as dexmethylphenidate HCL; this stimulant used to treat ADHD essentially saw the early onset of the summer decline when Covid-19 began to impact the US. The red line indicates the ramp up of Covid-19 cases and response in the US.

**Fig 3:**
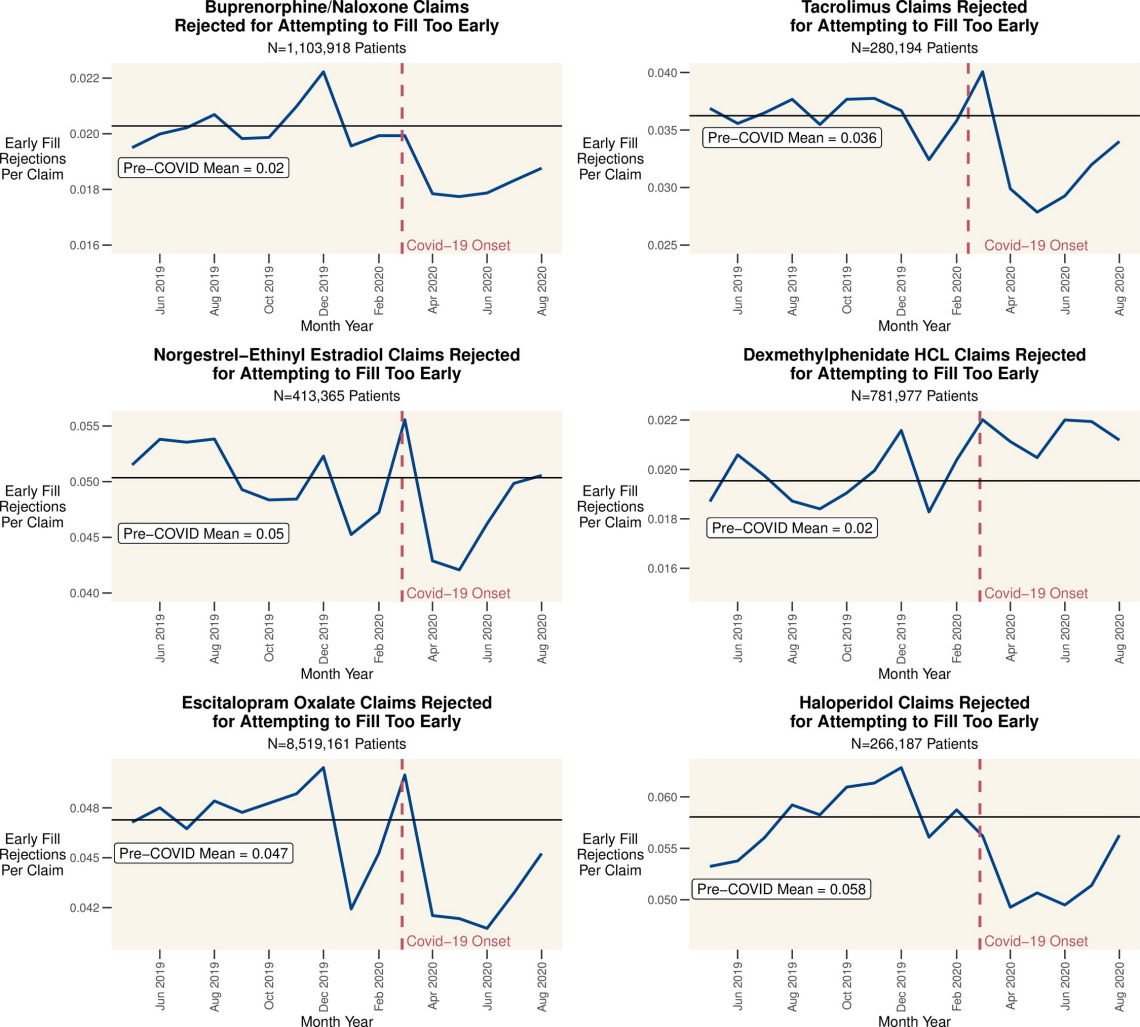
Proportion of total rejected for attempting to fill too early by month. Proportion of claims rejected for attempting to refill too early. This is calculated by dividing the number of claims rejected with NCPDP Code 79 –Early Refill by the total number of claims for the drug. Pre-COVID means are calculated through February 2020. The red line indicates the ramp up of Covid-19 cases and response in the US.

We first consider model free evidence. In Fig 1 we graph the total number of approved claims by month for all patients on the complete population of drugs indexed by SH. Fig 2 charts the total days of supply approved by month for our representative drugs. Fig 3 charts the proportion of claims rejected for attempting to refill too early, by drug, over time. Distinct peaks followed by dramatic and even larger declines in the prescribing of many drugs are evident from these simple crosstabulations. This suggests that while some patients pre-emptively filled their prescriptions, there was also a subsequent drop in refills by other patients which dwarfs this pre-emption.

### Statistical analysis

The primary dependent variable is *Discontinuation* of medication. This is determined by examining if there is a sufficient Days of Supply (DOS) dispensed to cover a given month. Using DOS instead of number of claims, pills, or milligrams allows the comparison of patients with different dosages and allows for a patient’s dosage to change. We evaluate each patient on a per-month basis, conditional upon whether they have enough supply to last through a given month (based on the DOS received over the previous 6 months). In doing so, we use a “28-day month” to allow for patients who receive fills in 7-day increments (e.g. 7, 14, 28) and to allow for occasional non-adherence (e.g. missed days). Aggregating patient activity at the monthly level smooths out noise in fill patterns and is a conservative approach because it prevents patients who are a few days late refilling from being erroneously classified as having discontinued. A visual inspection of the data indicates that most patients were either clearly adherent, filling 30 DOS per month, or clearly not, 30 DOS filled per month repeatedly and then stopping altogether. To screen out patients trialing a drug, or using it for an acute condition, we exclude patients that have not received at least one 54-day supply in a 60-day period. This cut point yields a natural break in the data, allows for 7/14/28-day refills to comprise a month, and will not erroneously capture patients who may miss occasional doses. For the focal drugs, we include all formulations under a given generic name, allowing patients to receive drugs from different manufacturers or have a dosage change. A detailed flow chart of claims included is in S1 Fig.

A conditional likelihood linear probability model (LPM) with robust standard errors clustered at the patient level is used to assess the likelihood of drug discontinuance after Covid-19 effects started. While non-linear estimations like Logits are typically preferred when modeling dichotomous outcomes, the LPM offers a tractable and interpretable solution which yields estimates consistent in terms of direction and size [10, 11]. Formally (Eq 1):

$$Discontinue=\beta_{0}+\beta_{1}\times PostCOVID+\tau_{i}$$ (1)

where *Discontinue* is the probability of a patient running out of a previous fill without refilling in a given month, *PostCOVID* is a 0/1 indicator for whether the month was after March 2020 (when the National Emergency was declared and the bulk of the Covid-19 impacts like lockdowns affected the US) [2]. *τ_i_* are patient-level fixed effects. *β*_0_ is a constant that lacks a generalizable interpretation due to the patient-level fixed-effects; *β*_1_ is the average change of the probability of a patient discontinuing use of their medication after Covid-19. To ensure robustness, we repeated the analysis with a logistic regression which yielded qualitatively similar results. We evaluate discontinuation of therapy by considering the patient’s claim history and assessing whether they had enough days of supply to last through a given month. In doing so, we consider all claims in the six months preceding the given month. Our metric captures intentional discontinuations of medication (e.g., at the prescriber’s direction), forced discontinuations (e.g., due to financial considerations), and unintentional discontinuations (e.g., due to forgetting). We also evaluate the total DOS of medication dispensed to a patient in a given 90-day period because of the different refill patterns of patterns (e.g., 30/60/90-day refills). This metric is more informative than evaluating the average quantity dispensed in any given month. Finally, we consider a patient to have “stocked up” during a month if they filled at least 60 DOS in a given month. Data was queried using Snowflake SQL Cloud Data Platform, visualizations were created in R version 4.0.2, and regression was completed in Stata/MP 16.1.

## Results

### Total prescriptions dispensed

There were 543.7 million prescription drug claims approved in March 2020, more than in any previous month. As seen in Fig 1, there is then a steep decline in subsequent months, with August, 2020 showing a 12.05% drop compared with August, 2019. As some of this decline may be attributed to diminished need due to social distancing, and thus diminished non-Covid-19 infections, we next focus on chronic medications being used for therapeutic purposes whose need is not expected to decline post-pandemic.

### Dispensing patterns of key drug classes and early refill claims rejections

The ramp-up of Covid-19 cases and response (e.g. lockdowns) in the United States began in March, 2020. Concurrently, there was an increase in claims (exceeding even the typical January surge) and days of supply (DOS) dispensed to patients. Indeed, March, 2020 saw the all-time high number of claims in a month for many drugs. Fig 2 shows the total DOS dispensed for six representative drugs. Most drugs follow a similar pattern, with a large stock-up in March followed by an eventual decrease in fills. Results for additional drugs are in S2 Fig.

The rejection of patient refills is also worth considering. Ordinarily, patients cannot refill prescriptions at will because payers reject the claim if the patient should still have enough medication on hand. These rejections are captured in the claims data by National Council for Prescription Drug Programs (NCPDP) Reject Code 79 –Early Refill. To support patients ahead of stay-at-home orders, many US states and payers relaxed rules regarding when patients could refill prescriptions. Fig 3 shows the claims rejected for attempting to refill too early as a percentage of total monthly claims, which declined for most products. Taken together, Figs 2, 3 provide consistent evidence that patients were attempting to, and permitted to, fill prescriptions early and stock up on their medication.

### Changes in quantities of drug accessed and likelihood of discontinuing use

Despite the increased quantities of drugs dispensed at the beginning of the Covid-19 pandemic, the question of whether patients were able to maintain access to chronic therapies is outstanding. Therefore, we model the impact of Covid-19 on two characteristics of drug utilization. First, the DOS the patient received in a given rolling 90-day period. This measure captures how much of a drug the patient had access to at a given time. Intuitively, having too much or too little is not ideal. Too much increases the chances of abuse, loss, or expiration while too little increases the chances of running out or withdrawal. Second, we model the within-person change in likelihood of discontinuing use of the drug. While some level of drug discontinuation is expected (e.g., due to loss of insurance, switching to another drug or completion of therapy), a large increase in discontinuation—absent another reason to stop use—raises concerns. Table 1 contains the results for the analysis of likelihood of discontinuing the drug. Table 2 presents the average number of active patients, the number of patients who appear to have stocked up by filling more than 60 DOS in March, and an estimate of the number of patients impacted by changes in the likelihood of discontinuing medication use. S3 Table contains the results for the analysis of DOS dispensed per 90-day period.

**Table 1:** Impact of Covid-19 on probability of discontinuing therapy: Conditional likelihood linear probability model analysis.

***Class***	***Addiction***	***Immunosuppression***	***Hormonal Contraceptive***
**Drug**	**Buprenorphine/Naloxone**	**Tacrolimus**	**Norgestrel-Ethinyl Estradiol**
** **	Coeff B	Coeff B	Coeff B
	(95% CI)	(95% CI)	(95% CI)
PostCOVID	-0.0059	-0.0015	0.0062
	(-0.0062 to -0.0055)	(-0.0019 to -0.0012)	(0.0059 to 0.0065)
	p<0.001	p<0.001	p<0.001
N Patients	N = 828,913	N = 280,444	N = 370,492
***Class***	***ADHD (Stimulant)***	***SSRI***	***Antipsychotic***
**Drug**	**Dexmethylphenidate HCL**	**Escitalopram Oxalate**	**Haloperidol**
	Coeff B	Coeff B	Coeff B
	(95% CI)	(95% CI)	(95% CI)
PostCOVID	0.0284	0.0057	0.0149
	(0.0279 to 0.0289)	(0.00561 to 0.00578)	(0.0141 to 0.0157)
	p<0.001	p<0.001	p<0.001
N Patients	N = 599,251	N = 7,633,955	N = 166,074

The PostCOVID coefficient estimates the average change in a patient’s probability of discontinuing the Post-Covid period compared to the Pre-Covid period. A positive coefficient for PostCOVID indicates that patients were more likely to discontinue the medication after Covid-19 while a negative term indicates patients are less likely to discontinue use. We include all generic and branded formulations in all strengths for the listed drugs. We include patients in the analysis only during the time that they demonstrate a pattern of active prescriptions, but results were consistent across a wide range of model specifications. For each case, we compute a linear probability model with fixed effects and clustered standard errors at the patient level; the fixed effects specification implies the constant term does not have an interpretation so it is not reported here for parsimony. The fixed effects help adjust for unobservables, but results were consistent when available controls for gender, ethnicity and household income were included instead. Results using a logistic regression model were qualitatively consistent for all drugs.

**Table 2:** Patient pool and changes in discontinuation.

*Class*	Average Number of Active Patients^a^	Extra Patients “Stocking Up” in March 2020^b^	Total	Estimated Discontinuations Resulting from Covid-19^d^	Avg Change in New Monthly Patients^e^
Drug			Discontinuations Post-Covid (March-Aug 2020)^c^		
***Addiction***	520,407	27,145	179,835	-20,531	-7,933
**Buprenorphine/**					
**Naloxone**					
***Immunosuppression***	200,773	20,161	27,893	-1,922	-1,450^f^
**Tacrolimus**					
***Hormonal Contraceptive***	240,740	6757	26,959	8,681	-2,771
** Norgestrel-Ethinyl Estradiol**					
***ADHD (Stimulant)***	358,472	7,980	148,426	54,777	-15,335^g^
** Dexmethylphenidate HCL**					
***SSRI***	4,511,608	279,061	897,621	164,867	-60,011
**Escitalopram Oxalate**					
***Antipsychotic***	82,658	5,734	30,440	8042	91
** Haloperidol**					

This table represents the potential scope of the impact of Covid-19 on medication adherence. These numbers represent only patients on these six drugs; the impacts are obviously magnified across all drugs and therapies. As seen in this table and the other figures, the exact impact of Covid-19 on a particular patient is uncertain: some patients stocked up more than normal, while others were more likely to discontinue use. The effects are heterogeneous across therapies as well.

^a^This is the number of patients demonstrating a pattern of actively filling prescriptions (beyond a single month or a trial of the drug). It is calculated by averaging the number of active patients from September 2019-February 2020 (pre-Covid months).

^b^This is the number of extra patients who “stocked up” on their medication in March, 2020. It is calculated by comparing the number of patients who filled prescriptions for 60+ DOS in March, 2020 against the average number of patients filling prescriptions for 60+ DOS in September 2019-February 2020.

^c^This represents the total number of patients discontinuing the medication from March 2020-August 2020. Some level of discontinuations is expected (e.g. changing to a different therapy).

^d^This is calculated by multiplying the linear probability model coefficients presented in Table 1 by the total number of active patient-months post-COVID; conceptually, it is the sum of exposing the active patients to the “change in likelihood of discontinuing.” Even small increases in the likelihood of discontinuing (e.g. a fraction of a percentage point) implies that thousands of additional patients will discontinue use. Note that in each case, it is only a fraction of the total discontinuations that are potentially attributable to Covid; however, it is a sizeable fraction. This leads us to believe that our results are plausible; even if estimates are off by an order of magnitude or more, tens of millions of patients are impacted across all drug categories.

^e^This is calculated by comparing the average number of new patients per month starting therapy in March 2020 –August 2020 against September 2019 –February 2020. Any patient with an approved claim is included in this analysis.

^f^The total number of transplants performed will be the primary driver of this number; access to immunosuppression is normally not a primary consideration. There will be some assessment of a patient’s access/coverage at the time of listing for transplant, but other factors (especially finding a match) are more significant. Live donor transplants were essentially halted in March 2020.

^g^See S3 Fig for additional discussion on seasonality.

Buprenorphine/naloxone (in strips or tablets) demonstrate an increase in claims dispensed from March through August, 2020 (Fig 2), and a reduction in rejections for early refills (Fig 3). From March, 2020 forward, patients filled claims for 1.04 more DOS (95% CI: 0.90 to 1.18, p<0.001) on average, indicating some patients had access to more of the drug than before, and the probability of discontinuing the drug decreased by 0.59% on average (95% CI: 0.55% to 0.62%, p<0.001).

Patients on tacrolimus, often closely managed by a transplant team, saw a sharp increase in prescriptions dispensed in March 2020. On average, the likelihood of discontinuing therapy decreased 0.15% (95% CI: 0.12% to 0.19%, p<0.001) and the DOS filled per 90-day period increased by 5.58 days (95% CI: 5.30 to 5.87, p<0.001) after March.

The remaining drugs we evaluated showed an increased likelihood of discontinuation from March forward. On average, patients taking the hormonal contraceptive norgestrel-ethinyl estradiol filled prescriptions for 2.81 fewer DOS (95% CI: 2.63 to 3.00, p<0.001, p<0.001) per 90-day period; likelihood of discontinuing increased 0.62% (95% CI: 0.59% to 0.65%, p<0.001). Further, the number of new patients starting the drug was down 21.4% on average from March, 2020 through August, 2020, as compared with the Pre-Covid months (September, 2019 to February, 2020).

One drug that did not see any increase in March is dexmethylphenidate HCL, a stimulant used to treat ADHD primarily in adolescents and young adults (mean Patient Age is 14.9 ± 10.8 years). Patients taking dexmethylphenidate HCL filled prescriptions for 1.79 fewer DOS (95% CI: 1.59 to 1.99) and were 2.84% more likely (95% CI: 2.79% to 2.89%, p<0.001) to discontinue use on average. Moreover, in addition to impacts on the existing patient pool, there were 45.8% fewer new patients each month on average once Covid began, as compared with the pre-Covid period.

The average patient on the SSRI escitalopram oxalate was 0.570% more likely (95% CI: 0.561% to 0.578%, p<0.001) to discontinue use of the drug compared with the pre-Covid period. Further, there were 17.6% fewer new patients per month on average after Covid. This should be concerning given that the CDC has reported that rates of the symptoms of anxiety and depression were “considerably increased” in April to June of 2020 when compared with the same months in 2019, so it seems unlikely that there were fewer patients needing treatment overall [12].

There was not an appreciable change in the average DOS filled by patients on the antipsychotic haloperidol, but the likelihood of discontinuing the drug increased by 1.49% on average (95% CI: 1.41% to 1.57%, p<0.001) compared to the pre-Covid period, but there was not an appreciable change in the number of new patients.

## Discussion

Results indicate a typical annual surge in prescriptions in January, 2020, followed by an unexpected surge in demand in March, 2020 as news of Covid-19 spread across the US and people began to anticipate stay-at-home orders. After this surge, there was a steep decline in overall prescriptions. Our results demonstrate that during the Covid-19 pandemic, some patients accessed extra medication and were potentially more adherent, while others were more likely to discontinue their medication.

The two drugs resistant to increased discontinuation, tacrolimus and buprenorphine/naloxone, are both generally administered with significant oversight to specific cohorts of patients. Buprenorphine/naloxone requires prescribers to acquire a waiver to provide medication assisted treatment (MAT). Because of this oversight, patients on this drug are more closely managed by their physician, which may have contributed to the diminished discontinuation rate. Similarly, patients taking tacrolimus are closely managed by their transplant team. These results are encouraging, indicating that existing patients treated in structured programs were not overtly disrupted by the pandemic.

However, patients taking the hormonal contraceptive norgestrel-ethinyl estradiol, the SSRI escitalopram oxalate, the antipsychotic haloperidol, or the stimulant dexmethylphenidate HCL were more likely to discontinue use post-Covid. As the estimates generated by a linear probability model are absolute (instead of relative) percentages, the estimates imply that hundreds of thousands of patients were affected just across the six therapies we evaluated. Discontinuing hormonal contraceptives has implications for individuals and families managing reproduction. Abruptly discontinuing an SSRI or antipsychotic can lead to withdrawal symptoms and have wide ranging implications for mental and ultimately physical health in a time when suicidal ideation increased overall [12, 13].

Across all drugs highlighted, except haloperidol, there was a decrease in the number of new patients initiating therapy. For example, the number of new patients initiating buprenorphine/naloxone therapy was down 18.8% on average compared with the pre-Covid months, indicating that new patients may not have been able to access treatment for opioid use disorder (See S4 and S5 Figs); even as the opioid epidemic continues to ravage communities and burden emergency departments across the country [14].

In contrast, findings indicate that new patients could initiate therapy with the antipsychotic haloperidol during the time studied. This may be because haloperidol is often initiated or restarted during inpatient hospitalizations for diseases involving overt psychosis (e.g. schizophrenia). And while it is an established antipsychotic which sees significant use in resource limited settings, there is higher pre-existing concern about treatment discontinuation for first-generation antipsychotics like haloperidol than second-generation antipsychotics [15], meaning first-generation antipsychotics are often turned to when a second-generation antipsychotic has been unsuccessful [16]. It is therefore worth considering whether similar patterns are observed with newer therapies. We thus replicate the analysis for patients taking quetiapine (a second-generation antipsychotic). Similar to haloperidol, patient prescriptions were filled for more DOS of quetiapine, and discontinuation increased Post-Covid (0.64% increase in likelihood, 95% CI: 0.63% to 0.65%, p<0.001). Yet, unlike haloperidol, fewer new patients were initiated on quetiapine. This underscores our finding that the medication access patterns during the early months of the Covid-19 pandemic vary widely across settings, therapies, and patients.

It is also worth noting that Dexmethylphenidate HCL shows a strong seasonal usage pattern (see S4 Fig), typically showing a 20–25% decline in prescription fills in June followed by a corresponding resurgence in September. For many years, the American Academy of Family Physicians advised that children “whose ADHD symptoms predominantly affect schoolwork” to take summer drug holidays, though the practice is not universal [17, 18]. Covid-19 seems to have ushered in an early decline for dexmethylphenidate HCL utilization beginning in March 2020 instead of June when normally anticipated. It also appears that some patients began a drug holiday when most learning moved online, and significant workforce disruptions began. It is unclear whether this was an intentional choice made because learning and/or working in the less structured home environment did not require stimulant medication or whether individuals could not access the medication due to inability to reach their provider or pharmacy. Likewise, young adults may have lost the very jobs they required the medication to adequately perform. Ultimately, the apparent shift in drug utilization could have long lasting implications for patients’ education, development, and careers and warrants further attention.

It further bears note that while this is, to the best of our knowledge, the first study to evaluate Covid-related access issues across a range of therapies using a comprehensive dataset of US patients, our method is similar to Esposti et. al. [19]. This study of Italian patients also identifies increased rates of “failure to refill” prescriptions for lipid-lowering and biologic therapies. Findings of the potential for increased adherence (as we observed for patients taking tacrolimus and buprenorphine/naloxone) are not without precedent either. A single-center study of patients receiving growth hormone therapy did not identify Covid-related increases in non-adherence (though they did highlight the need for increased caregiver diligence due to supply issues) [20], and increased adherence post-Covid has been reported in a study of 7578 asthma and COPD patients using an inhaler [21]. However, this could be related to patient vigilance in the setting of a pulmonary pandemic. The diversity of the literature on this topic reinforces that therapies should be studied independently to accurately identify access issues and propose strategies to minimize impacts to patients during future global disruptions.

### Study strengths and limitations

A key strength of this study is the access to one of the most comprehensive databases of US healthcare delivery. This is particularly important in evaluating the effects of Covid-19 because many patients have either been forced to switch pharmacies or lost coverage. Nevertheless, this work is subject to several limitations which offer rich opportunities for future work.

First, we cannot directly observe patient adherence (i.e. whether patients are taking their medication) or the reasons for discontinuation (e.g. loss of coverage, impact of stay-at-home orders). While the scale of the analysis far exceeds what is possible in adherence studies that rely on pill counts, MEMS caps, or patient journals [22], and represents a conservative estimate because prescription counts represent a lower bound, this is simply a limitation on secondary data. Second, we review a limited set of drugs and trends. As discussed above, the observed patterns for a given drug are not determinative of another drug’s patterns (as is demonstrated with haloperidol and quetiapine). A valuable extension of this paper will be to examine how drug within a given class of interest trend together. Similarly, patient-centered work will help tease apart the motivations and obstacles to access during this time period.

## Conclusions

This investigation yields three key findings for practitioners and policy makers. First, there has been a significant decline in new patients starting therapy across a number of key drug classes during the ongoing pandemic. Second, many existing patients were able to fill their prescriptions for chronic medications during the early months of the Covid-19 pandemic. Finally, for many drugs, a greater number of patients were likely to discontinue use. Encouragingly, patients were less likely to discontinue opioid use disorder therapy (buprenorphine/naloxone) and immunosuppression (tacrolimus). This resilience to disruption by the pandemic may be attributed to patients being closely managed as a cohort (e.g., a primary care doctor’s MAT patients or a center’s post-transplant patients). Ultimately, the net effects of Covid-19 on a particular patient’s medication adherence are uncertain. Providers would do well to inquire about adherence disruption during 2020 and 2021 and provide prompt, nonjudgmental, re-initiation of medications where necessary.

### Data access

The data, technology, and services used in the generation of these research findings were supplied by the COVID-19 Research Database partners, who are acknowledged at https://covid19researchdatabase.org/. Because this dataset contains insurance claims which could potentially identify individual patients, we are legally prohibited to post the dataset publicly. Researchers wishing to replicate or extend this work may obtain access, subject to DUA, by contacting the COVID-19 Research Database.

## Supporting information

S1 Fig Inclusion process for data analysis. (PDF)

S2 Fig Claims totals and early fill rejection patterns for additional drugs analyzed. (PDF)

S3 Fig Dexmethylphenidate HCL long-term seasonality. (PDF)

S4 Fig New patients by month—12 months from September 2019 to August 2020. (PDF)

S5 Fig Adverse event claims for opioid overdose. (PDF)

S1 Table Drug shortage status for representative drugs. (PDF)

S2 Table Summary statistics for symphony claims database: May 2019 through Aug. (PDF)

S3 Table Impact of Covid-19 on days of supply dispensed per 90-day window. (PDF)
