# Supplementary figures and images for: Patient access to chronic medications during the Covid-19 pandemic: Evidence from a comprehensive dataset of US insurance claims
Source: PLoS One. 2021 Apr 1;16(4):e0249453. doi: 10.1371/journal.pone.0249453 (PMC8016279; doi:10.1371/journal.pone.0249453)

## S1 Fig. Inclusion Process for Data Analysis

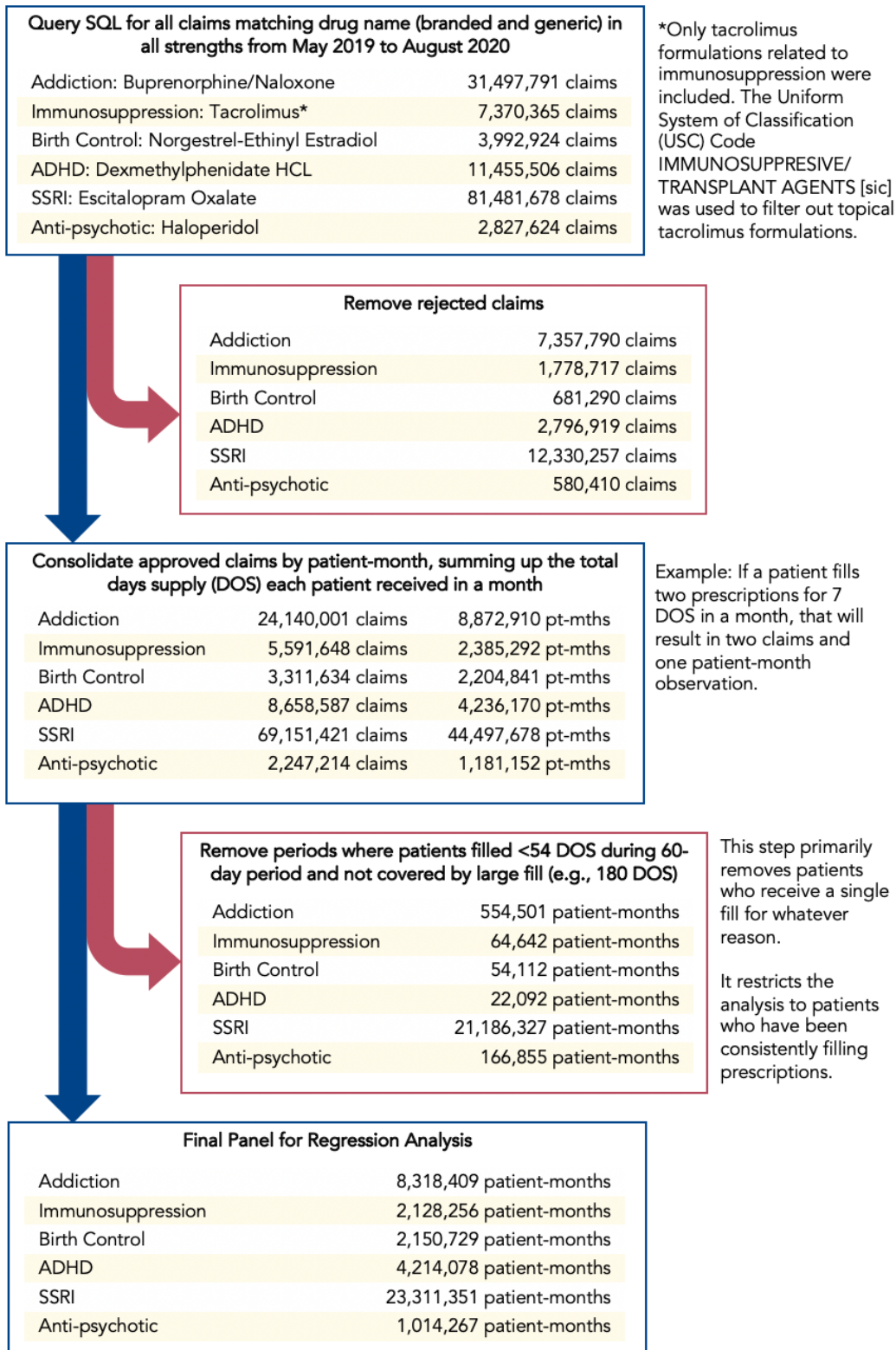

Supplement: S1 Fig — (PDF) [file pone.0249453.s001.pdf]
