# Supplementary material for: Patient access to chronic medications during the Covid-19 pandemic: Evidence from a comprehensive dataset of US insurance claims
Source: PLoS One. 2021 Apr 1;16(4):e0249453. doi: 10.1371/journal.pone.0249453 (PMC8016279; doi:10.1371/journal.pone.0249453)
Supplement: S2 Fig — (PDF) [file pone.0249453.s002.pdf]

# S2 Fig. Claims Totals and Early Fill Rejection Patterns for Additional Drugs Analyzed

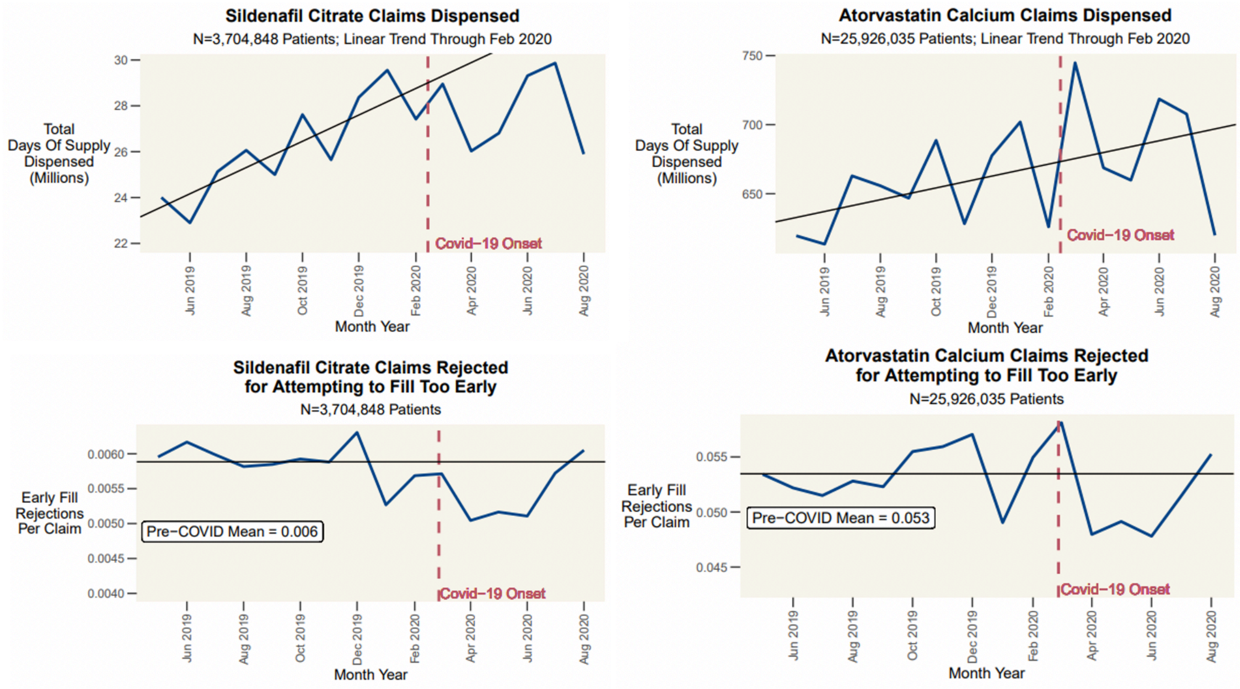


s2 Figure Notes: Claims for sildenafil citrate (used primarily for erectile dysfunction) declined significantly in the early months of Covid-19. The analysis for sildenafil citrate is less clear than many other drugs since it not necessarily used daily. Some prescribers indicate that e.g., 6 tablets is a 30 day supply while others indicate 6 tablets is a 6 day supply (not necessarily intended for them to be used on sequential days). Thus, evaluating discontinuation from claims data is less clear. However, the clear decline in fills has implications for relationships and mental health.

The predominant statin, atorvastatin calcium, saw the most claims and most drug dispensed ever in March 2020. Most patients on the statin have maintained access, and results from the fixed effect linear probability model estimate patients were 0.492% less likely (95% CI: 0.488% to 0.495%) to discontinue use of atorvastatin calcium after Covid-19 compared with pre-Covid. This low rate of assumed Discontinuation may be driven by the large “stock up” in March 2020; it is uncertain whether the rate of Discontinuation will remain low after patients use up their current stocks of medication.
