# Supplementary material for: Patient access to chronic medications during the Covid-19 pandemic: Evidence from a comprehensive dataset of US insurance claims
Source: PLoS One. 2021 Apr 1;16(4):e0249453. doi: 10.1371/journal.pone.0249453 (PMC8016279; doi:10.1371/journal.pone.0249453)
Supplement: S3 Fig — (PDF) [file pone.0249453.s003.pdf]

S3 Fig. Dexmethylphenidate HCL Long-term Seasonality

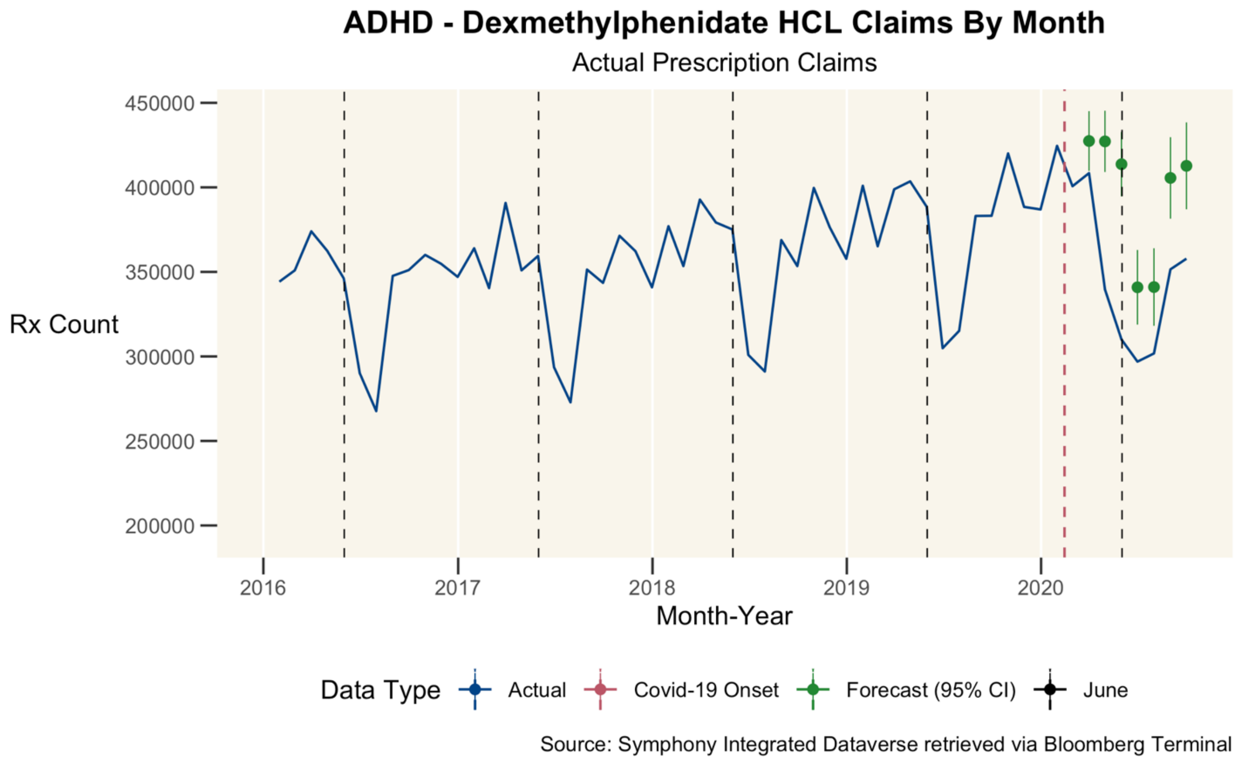

S3 Figure Notes: Prescription claims from the Symphony Integrated Dataverse providing aggregate data on claims in the US. This dataset is not as comprehensive as the patient-level claims data used in our main analysis, covering about 74% of the prescriptions dispensed. However, this data is available going back to 2009 allowing for excellent analysis of seasonality. Medications used for ADHD show distinct seasonality patterns with a slight decline in May followed by a sharp decline in June each year. The June months are denoted with the black dotted lines; the red dotted line represents the onset of Covid-19.

In this analysis, we developed an ARIMA model of utilization which accounts for seasonality, using data from January 2009 (earliest available) through February 2020 (Covid-19 onset). The forecasts from this model are shown as green dots with 95% CI error bars. The onset of Covid-19 essentially “moved up” the decline in dexmethylphenidate HCL prescription claims to March and substantially reduced the typical reuptake in August and September. The consequences for children who would have otherwise been on the drug are uncertain.
