# Supplementary material for: Patient access to chronic medications during the Covid-19 pandemic: Evidence from a comprehensive dataset of US insurance claims
Source: PLoS One. 2021 Apr 1;16(4):e0249453. doi: 10.1371/journal.pone.0249453 (PMC8016279; doi:10.1371/journal.pone.0249453)
Supplement: S4 Fig — (PDF) [file pone.0249453.s004.pdf]

S4 Fig. New Patients by Month - 12 Months from September 2019 to August

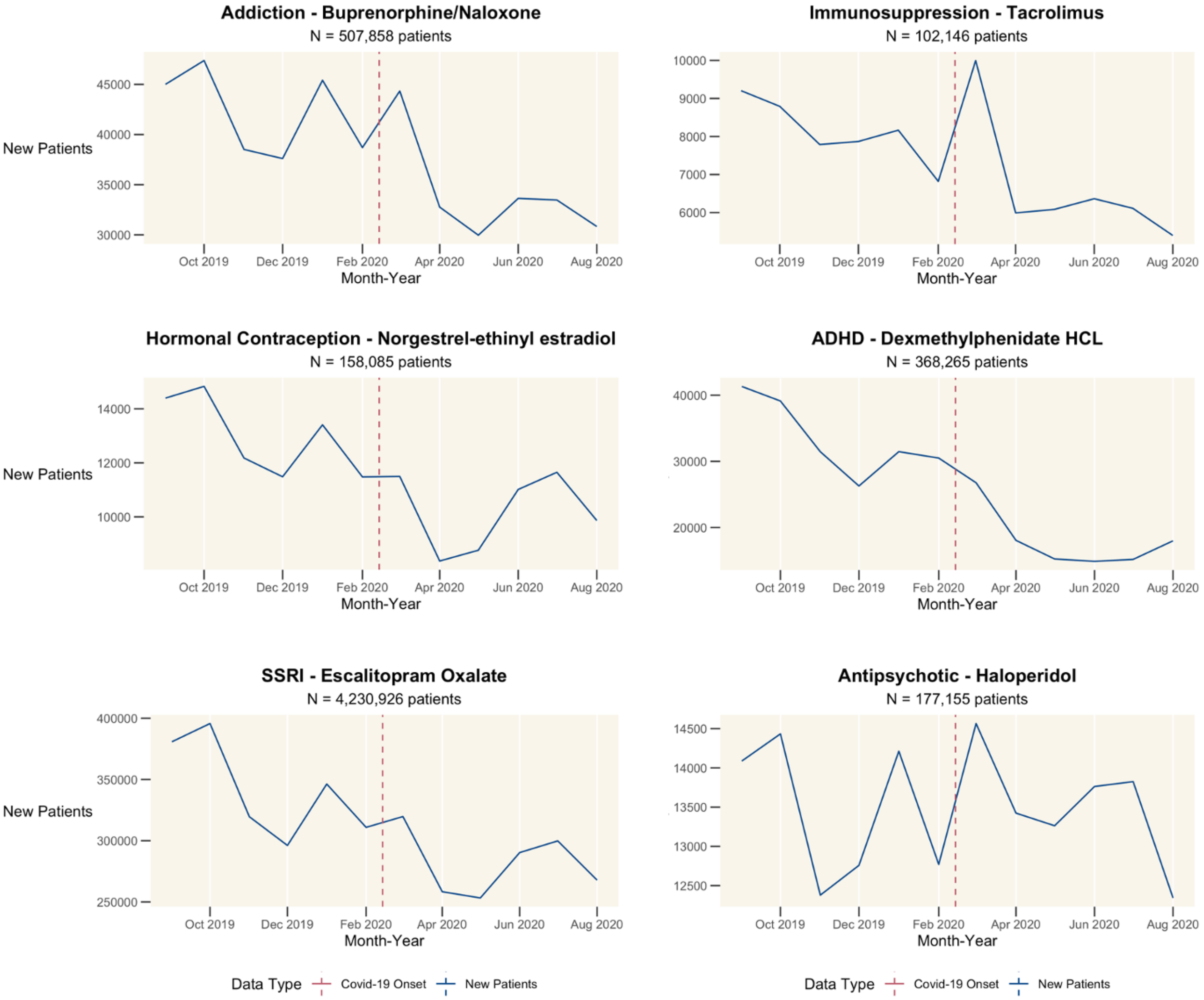

2020

S4 Figure Notes: The number of new patients starting on each drug by month from September 2019 through August 2020. Patients are counted as a "new patient" in the month their first claim is approved. This analysis includes all patients with at least one approved claim dispensed. The spike in new tacrolimus patients is unexplained by the data; those patients are primarily associated with diagnosis codes Z94.x related to transplant. It is partly explained by an increase in patients newly switching to tacrolimus from other immunosuppressants (e.g., everolimus, sirolimus and cyclosporine). Due to the unique payer dynamics related to transplant, most kidney transplants are covered by Medicare Part B regardless of patient age. It is possible that SH gained access to additional patient data from a Medicare Part B payer. The within-patient discontinuation likelihood analysis is not compromised by these additional observations in the way a survival analysis would be.
