# Supplementary material for: Patient access to chronic medications during the Covid-19 pandemic: Evidence from a comprehensive dataset of US insurance claims
Source: PLoS One. 2021 Apr 1;16(4):e0249453. doi: 10.1371/journal.pone.0249453 (PMC8016279; doi:10.1371/journal.pone.0249453)
Supplement: S5 Fig — (PDF) [file pone.0249453.s005.pdf]

S5 Fig. Adverse Event Claims for Opioid Overdose

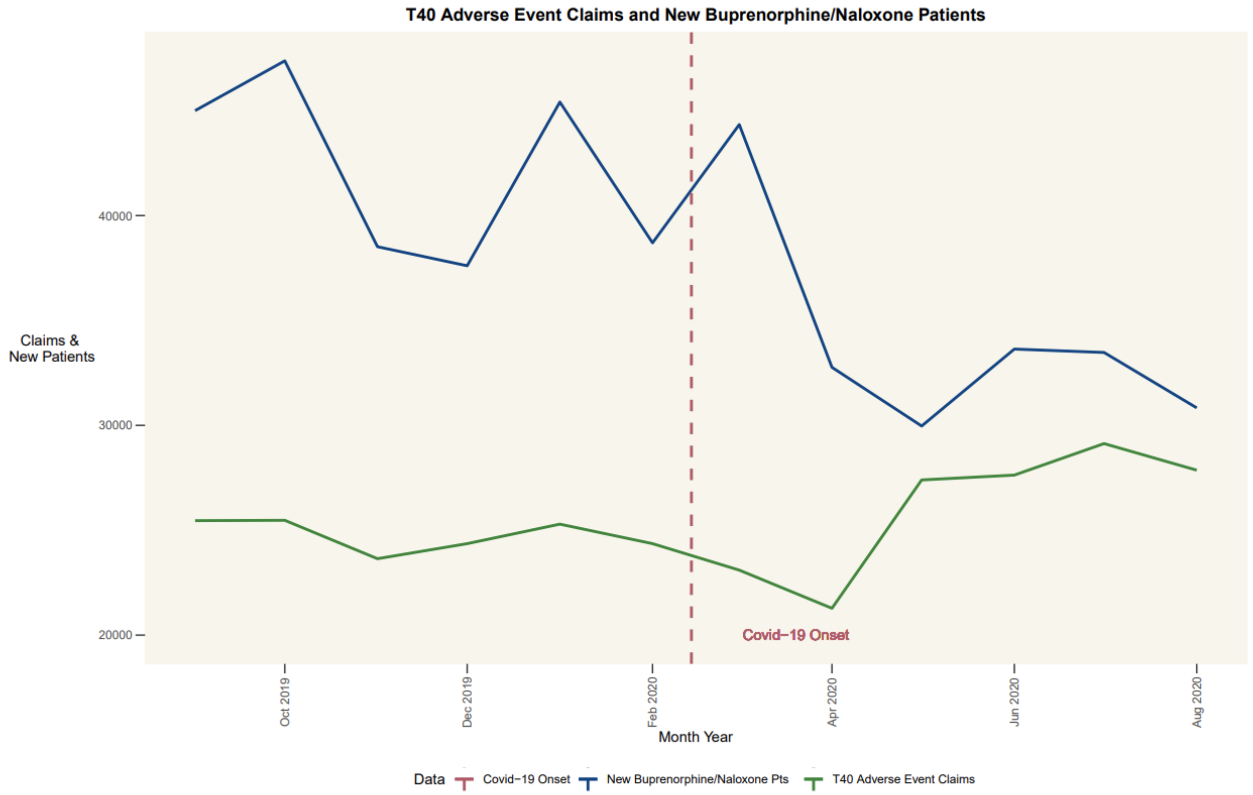

S5 Figure Notes: After a slight initial drop, the post-Covid period saw an increase in the number of claims for opiod-related adverse events (shown on the lower line in green). The number of new patients receiving buprenorphine/naloxone declined 18.8% on average in the months post-Covid compared with the six months prior to Covid.
