# Supplementary material for: Patient access to chronic medications during the Covid-19 pandemic: Evidence from a comprehensive dataset of US insurance claims
Source: PLoS One. 2021 Apr 1;16(4):e0249453. doi: 10.1371/journal.pone.0249453 (PMC8016279; doi:10.1371/journal.pone.0249453)
Supplement: S1 Table — (PDF) [file pone.0249453.s006.pdf]

S1 Table. Drug Shortage Status for Representative Drugs

S1 Table: Drug Shortage Status for Representative Drugs in Study	
Class<br>**Drug**	**Shortage Status**
Addiction<br>Buprenorphine/Naloxone	No current or resolved shortages reported
Immunosuppression<br>Tacrolimus	Some tacrolimus manufacturers and formulations (especially generic) have been on shortage since June 2019, but branded and extended release formulations have been available without interruption. Covid-19 has not apparently changed or exacerbated the shortage. At least one branded manufacturer has consistently advertised their uninterrupted supply throughout the shortage.
Hormonal Contraceptive<br>Norgestrel-Ethinyl Estradiol	No current or resolved shortages reported
ADHD (Stimulant)<br>Dexmethylphenidate HCL	No current or resolved shortages reported
SSRI<br>Escitalopram Oxalate	No current or resolved shortages reported
Antipsychotic<br>Haloperidol	One generic manufacturer discontinued haloperidol production in August 2018, but at least two major manufacturers have had haloperidol available.

S1 Table Notes: Drug shortages are a significant issue and are carefully tracked by the American Society of Health-System Pharmacists (ASHP). Drug shortage information is maintained by ASHP at https://www.ashp.org/Drug-Shortages and goes back to at least 2015. The focal drugs in our study did not experience new manufacturer shortages due to the Covid-19 pandemic. There could be still be local distribution issues impacting patients.
