# Supplementary material for: Patient access to chronic medications during the Covid-19 pandemic: Evidence from a comprehensive dataset of US insurance claims
Source: PLoS One. 2021 Apr 1;16(4):e0249453. doi: 10.1371/journal.pone.0249453 (PMC8016279; doi:10.1371/journal.pone.0249453)
Supplement: S2 Table — (PDF) [file pone.0249453.s007.pdf]

S2 Table. Summary Statistics

S2 Table: Summary Statistics for Symphony Claims Database: May 2019 through Aug 2020			
Class **Drug**	***Addiction*** **Buprenorphine/Naloxone**	***Immunosuppression*** **Tacrolimus**	***Hormonal Contraceptive*** **Norgestrel-Ethinyl Estradiol**
Total Claims	31,507,516	7,370,365	3,992,924
Rejected - Any Reason	7,359,995	1,778,717	2,796,919
Filled Claims	24,147,521	5,591,648	8,658,587
N Patients	1,103,507	280,194	8,195,895
Mean Age (SD)	40.1 (11.7)	52.9 (17)	40.1 (11.7)
Pct Female	43.1%	43.0%	99.5%
Class **Drug**	***ADHD (Stimulant)*** **Dexmethylphenidate HCL**	***SSRI*** **Escitalopram Oxalate**	***Antipsychotic*** **Haloperidol**
Total Claims	11,455,506	81,481,678	2,827,624
Rejected - Any Reason	2,796,919	12,330,257	580,410
Filled Claims	8,658,587	69,151,421	2,247,214
N Patients	760,743	8,195,895	266,081
Mean Age (SD)	14.9 (10.8)	45.3 (19)	51.9 (19.6)
Pct Female	33.9%	69.6%	48.1%

S2 Table Notes: Summary statistics across all patients in the Symphony Prescriptions Claims database with at least one claim for the listed drug. The analysis includes all branded and generic formulations of the drug in question in all strengths.
