# Supplementary material for: Patient access to chronic medications during the Covid-19 pandemic: Evidence from a comprehensive dataset of US insurance claims
Source: PLoS One. 2021 Apr 1;16(4):e0249453. doi: 10.1371/journal.pone.0249453 (PMC8016279; doi:10.1371/journal.pone.0249453)
Supplement: S3 Table — (PDF) [file pone.0249453.s008.pdf]

S3 Table. Impact of Covid-19 on Days of Supply Dispensed per 90-day Window

S3 Table: Impact of Covid-19 on Days of Supply Dispensed per 90-day Window

Class	***Addiction***	***Immunosuppression***	***Hormonal Contraceptive***
**Drug**	**Buprenorphine/Naloxone**	**Tacrolimus**	**Norgestrel-Ethinyl Estradiol**
	Coeff B (95% CI)	Coeff B (95% CI)	Coeff B (95% CI)
PostCOVID	1.04 (0.90 to 1.18) p<0.001	5.58 (5.30 to 5.87) p<0.001	-2.81 (-3.00 to -2.63) p<0.001
N Patients	N = 748,357	N = 286,758	N = 343,161

Class	***ADHD (Stimulant)***	***SSRI***	***Antipsychotic***
**Drug**	**Dexmethylphenidate HCL**	**Escitalopram Oxalate**	**Haloperidol**
	Coeff B (95% CI)	Coeff B (95% CI)	Coeff B (95% CI)
PostCOVID	-1.79 (-1.99 to -1.59) p<0.001	-0.041 (-0.085 to 0.004) p=0.073	0.03 (-0.34 to 0.40) p=0.877
N Patients	N = 550,783	N = 7,003,398	N = 147,792

S3 Table Notes: This analysis considers how much each average patient have received in the previous 90-day period. The constant term is the baseline average Days of Supply (DOS) dispensed to each patient, while the PostCOVID term represents any change (more or fewer DOS) the average patient receives over a 90-day period. We include all generic and branded formulations in all strengths for the listed drugs; we include patients in the analysis only during the time that they demonstrate a pattern of active prescriptions. For each drug, we compute a linear regression model with fixed effects and clustered standard errors at the patient level. See Supplementary Figure S1 for a diagram of which claims were included.
